# National Efficiency

**Published:** 1910-02

**Authors:** 


### National Efficiency.

Since the greatest of our national assets in the health and vigor of the American people, our efficiency must depend on national vitality even more than on the resources of the minerals, lands, forests and waters.

The average length of human life in different countries varies from less than twenty-five to more than fifty years. This span of life is increasing wherever sanitary science and preventive medicine are applied. It may be greatly extended.

Our annual mortality from tuberculosis is about 150,000. Stopping three-fourths of the loss of life from this cause, and from typhoid and other prevalent and preventable diseases would increase our average length of life over fifteen years.

There are constantly about 3,000,000 persons seriously ill in the United States of whom 500,000 are consumptives. More than half this illness is preventable.

If we count the value of each life lost at only $1700, and reckon the average earning lost by illness at $700 per year for grown men, we find that the economic gain from mitigation of preventable disease in the United States would exceed $1,500,000,000 a year. In addition we would decrease suffering and increase happiness and contentment among the people. This gain, or the lengthening and strengthening of life which it measures, can be secured through medical investigation and practice, school and factory hyggiene, restriction of labor by women and children, the education of the people in both public and private hygiene, and through improving the efficiency of our health service—municipal, State and National. The National government has now several agencies exercising health functions which only need to be concentrated to become co-ordinated parts of a greater health service worthy of the nation.—*From the Report of the National Conservation Commission.*

